# Facilitators and barriers to COVID-19 vaccine uptake among women in two regions of Ghana: A qualitative study

**DOI:** 10.1371/journal.pone.0272876

**Published:** 2022-08-17

**Authors:** Grace Frempong Afrifa-Anane, Reuben Tete Larbi, Bright Addo, Martin Wiredu Agyekum, Frank Kyei-Arthur, Margaret Appiah, Clara Opoku Agyemang, Ignatius Great Sakada

**Affiliations:** 1 Department of Environment and Public Health, University of Environment and Sustainable Development, Somanya, Ghana; 2 Regional Institute for Population Studies, University of Ghana, Legon, Ghana; 3 Department of Sociology and Social Work, Kwame Nkrumah University of Science and Technology, Kumasi, Ghana; 4 Institute for Educational Research and Innovation Studies, University of Education, Winneba, Ghana; 5 Department of Population, Family and Reproductive Health, Kwame Nkrumah University of Science and Technology, Kumasi, Ghana; KEMRI Wellcome Trust Research Programme, KENYA

## Abstract

Although COVID-19 vaccines are available, evidence suggests that several factors hinder or facilitate their use. Several studies have found gender differences in COVID-19 vaccine uptake, with women less likely to vaccinate than men in many countries, including Ghana. These studies, however, have primarily been quantitative. This study used a qualitative approach to examine the facilitators and barriers to vaccine uptake among women in Ghana. Using a cross-sectional descriptive qualitative research design, 30 women in the Greater Accra and Ashanti regions of Ghana were conveniently sampled and interviewed using a semi-structured interview guide. Fifteen (15) interviews were conducted in each region. The data were transcribed verbatim and analysed thematically using QSR NVivo version 10 software. Among the key factors that facilitate COVID-19 vaccination are the desire to protect oneself and family against COVID-19, education about COVID-19 vaccines, seeing others receive the COVID-19 vaccine, and vaccine being cost-free. On the other hand, long queues at the vaccination centres, fear of side effects, misconceptions about the vaccines, and shortage of vaccines were the main barriers against COVID-19 vaccination. The study results show that individual, institutional, and vaccine-related factors facilitate or hinder COVID-19 vaccination among women. Addressing these factors need continuous comprehensive health education, and ensuring vaccine availability at vaccination sites will improve women’s uptake of the COVID-19 vaccines.

## Introduction

COVID-19 remains a health and socio-economic threat to the world. As of 16^th^ May 2022, there had been 519,105,112 cumulative confirmed cases and 6,266,324 deaths globally [1]. Ghana recorded the first case of COVID-19 on 12^th^ March 2020. Following the increasing number of COVID-19 cases, the government of Ghana implemented a partial lockdown on 30^th^ March 2020 in two major cities: Accra and Kumasi. The partial lockdown affected the livelihood of persons residing in Accra and Kumasi, especially the vulnerable, making it difficult for them to adhere to the lockdown restrictions. Consequently, the lockdown was lifted on 20^th^ April 2020, due to economic hardship on the vulnerable despite the rising cases of COVID-19 [2,3]. In addition, the government of Ghana provided the necessary logistics and regulations to curb the spread of COVID-19, such as the provision of isolation centres and personal protective equipment (PPEs) to health workers, mandatory 14-day quarantine of travellers arriving in the country and tracing contacts of infected persons.

COVID-19 vaccines are considered a means of protection by curtailing the spread of COVID-19 and reducing its related hospitalization and mortality. There is no doubt that the COVID-19 vaccine has reduced infection rates, severe morbidity and thus saved many lives globally since its deployment in 2020 [4–6]. The coverage of the COVID-19 vaccine varies among countries but it is particularly low in Africa, which could be attributed to issues of vaccine development and production, allocation, affordability, and deployment [7]. Africa has the least number of persons vaccinated against COVID-19. As of 15^th^ May 2022, Africa had 19.60 persons vaccinated with at least one dose per 100 population compared to 78.89 and 85.76 in the Americas and Western Pacific, respectively [1]. In Ghana, the COVID-19 vaccination rate is moderate compared to other African countries. About 31.52 persons in Ghana have been vaccinated with at least one dose per 100 population compared with 67.50 in Morocco and 1.98 in the Democratic Republic of the Congo as of 15^th^ May 2022 [1]. Regarding COVID-19 related mortality, Ghana had recorded 1,445 deaths compared with 16,071 deaths in Morocco, 1,338 deaths in the Democratic Republic of the Congo and 273 deaths in Togo as of 16^th^ May 2022 [1]. The leading cause of the low vaccination coverage in Africa has been attributed to access to vaccines and associated logistics such as syringes. Low- and middle-income countries, particularly in Africa, depend mainly on donations through the COVAX facility to deploy vaccination programme [8].

In addition to access to vaccines and logistical problems, willingness to take the vaccine is a significant determinant of rollout rates. Vaccine hesitancy has prevailed at national and individual levels; doses of the Oxford–AstraZeneca vaccine delivered to the European Union at the beginning of the vaccination campaign were not administered [9]. News about the formation of blood clots among some population groups after receiving the AstraZeneca jab [10,11] was a primary concern for potential vaccine receivers. The ‘5C model of drivers of vaccine hesitancy’ developed based on research in industrialized countries highlights five main individual-level determinants of vaccine hesitancy. These are: (i) Confidence–trust in the safety and effectiveness of vaccines, the system that administers vaccination, and the motivation of individuals who decide on the need for vaccination; (ii) Convenience (or constraints)–reflects the structural or psychological barriers to the uptake of vaccination; (iii) Complacency–perceiving risks and level of threat of vaccine-preventable diseases low; (iv) Risk calculation–a deliberate assessment of the risks of infection and vaccination, to derive a decision; (v) Collective responsibility which denotes one’s willingness to protect others by getting vaccinated, through herd immunity [12,13]. In the United Kingdom, national cross-sectional surveys found that fear of the potential side effects is a significant cause for vaccine hesitancy [14,15].

There are reported socio-economic differentials in the vaccine uptake globally. For example, Wouters et al. [7] noted lower levels of acceptance of vaccines in Serbia, Lebanon, Croatia, France, and Paraguay but higher acceptance in Vietnam, Indian, China, South Korea and Denmark. In addition, black and minority ethnic groups are less likely to take the vaccine. Also, gendered differences in the uptake of the COVID-19 vaccine have been reported in many countries, including Ghana. Researchers have found that females are less likely to take the COVID-19 vaccine than males [16–20]. For instance, Schwarzinger et al. [21] examined COVID-19 vaccine hesitancy among persons aged 18–64 years in France using an online survey and found refusal significantly associated with being female, lower educational level, having no chronic condition, lower perceived severity of COVID-19, being at the middle age range (that is 18–64), and past vaccine non-compliance. Also, through a national cross-sectional survey in Qatar, Alabdulla et al. [22] reported that females had a higher likelihood of vaccine hesitancy than males. In Ghana, similar findings have been found among healthcare workers [16] and the general population [20,23,24]. The consistent relationship between gender and vaccine hesitancy necessitates a critical assessment of why it is the case. The literature suggests risk perception, misinformation, age, and educational attainment or a combination of these factors as moderators of the gender and vaccine hesitancy relationship [16,25,26].

Studies that have reported gendered differentials in vaccine hesitancy are mainly quantitative and have used cross-sectional surveys to predict the likelihood of vaccine uptake [16–18,22,24]. While quantitative models and predictions are essential for designing appropriate public health interventions to encourage vaccine uptake, the knowledge gap on why this gendered differential exist have not been well explained by these quantitative studies. These existing quantitative studies have been valuable in explaining patterns and predicting outcomes but have limited utility in explaining why these behaviours or choices do occur. Success in the COVID-19 vaccination campaign is contingent on understanding why people will or will not take the vaccine. This study, therefore, seeks to fill this knowledge gap in socio-demographic differences in COVID-19 vaccine uptake by using a qualitative approach to critically examine the facilitators and barriers to vaccine uptake among women in Ghana.

## Materials and methods

### Study design and sampling procedure

This study used a cross-sectional descriptive qualitative research design to interview women in the Greater Accra and Ashanti regions of Ghana using a convenient sampling procedure. The descriptive qualitative research design enable researchers to describe the experiences and perceptions of people [27]. We conveniently approached women in the Greater Accra and Ashanti regions, and those who were readily available and willing to be interviewed face-to-face were interviewed for the study. A convenient sampling procedure was used because it is less costly and time-consuming. Participants included in the study were non-pregnant women aged 18 years or older residing in the Greater Accra and Ashanti regions of Ghana.

### Study setting

Ghana has 16 administrative regions, and Greater Accra and Ashanti regions, the study areas, contain a little over one-third (35.3%) of the population living in Ghana [28]. Accra is the capital of the Greater Accra Region, and it is also the national capital of Ghana, while Kumasi is the capital of the Ashanti region. The Greater Accra region has a population of 5,455,692 persons, while the Ashanti region has 5,440,463 persons. It is worthy of note that the Greater Accra and Ashanti regions have been the main hotspots of COVID-19 cases in Ghana. The Greater Accra region is more urbanized (91.7%) compared to the Ashanti region (61.6%) [28].

The Ashanti region has more health facilities and critical health staff than the Greater Accra region [29]. The Ashanti Region has 1,659 health facilities compared to 1,087 in Greater Accra as of 2017. Specifically, the Ashanti region has 1,122 Community-based Health Planning and Services (CHPS), 25 district hospitals, 164 health centres, and 128 hospitals compared to 498 CHPS, 8 district hospitals, 40 health centres, and 99 hospitals in the Greater Accra region. Overall, the Ashanti Region has 13,438 critical health staff (such as community health nurses and enrolled nurses, among others) compared to 13,120 in the Greater Accra region. Also, Greater Accra has a lower doctor-to-population ratio (3,052 persons per doctor) compared to the Ashanti Region (6,888 persons per doctor) as of 2017 [29].

### Data collection

The data collection for the study was conducted over two months, starting from October 2021 to November 2021. In total, 30 in-depth interviews were conducted. Fifteen (15) interviews were conducted in Greater Accra and Ashanti regions, respectively, which enabled the researchers to achieve data saturation. Research has established that data saturation can be achieved with twelve interviews [30]. The authors received a day-training on the interview guide and how to ask the questions in Akan (Niger-Congo language spoken in Ghana). The fifth, sixth, seventh and eighth authors conducted in-depth interviews in the Akan and English after explaining the purpose of the study to participants. On average, the interviews lasted 40 minutes. The in-depth interview guide administered to the participants covered topics on their socio-demographic characteristics, COVID-19 experience, COVID-19 vaccination, motivation to accept COVID-19 vaccine, and barriers to accept COVID-19 vaccine (See S1 File). The interview guide was piloted with women in Greater Accra and Ashanti regions to assess how they understood the questions and probes. In addition, the pilot study helped the researchers to revise the interview guide for better understanding. All in-depth interviews were audio-recorded, and the eighth author transcribed all interviews conducted in Akan verbatim into English. The other co-authors reviewed the transcripts for accuracy.

Participation in the study was voluntary, and participants gave their written consent before being interviewed. Face-to-face interviews were administered at the homes of participants and the authors ensured privacy throughout the interview. The study observed all ethical considerations concerning human subjects. Ethical approval for the study was obtained from the Ethics Committee of the University of Environment and Sustainable Development, Somanya—Ghana (APP/RSC/0001). All participants were thanked after they were interviewed to show appreciation for their time.

### Data analysis

The data were analysed thematically using QSR NVivo version 10 software. Attride-Stirling’s [31] conceptualized a thematic analysis and explained that it systemizes text extraction from larger textual data. The data analysis process involved reading the transcripts to familiarise oneself with the text after which, the text was coded. Both deductive and inductive codes were generated. Deductive codes were derived from literature and theory, while inductive codes emerged from the transcripts. All authors were involved in the data analysis. First, all authors read the transcripts to identify the codes and themes. Second, after reviewing the transcript, all authors provided feedback on identified codes and themes. Third, all authors then agreed on the codes and themes.

Using the constant comparison principle, similar codes were put together into one theme, which aided in getting the different levels of themes; basic, organizing and global themes. Basic themes are the lowest order theme. They are simple premises characteristic of the data and make little sense beyond its immediate meaning. For the basic themes to make more meaning, they have to be clustered together on similar issues. Organizing themes are middle-order themes that summarise the assumptions of a group of basic themes. They are more revealing of what is going on in the texts. Global themes were then developed from the organizing themes.

## Results

### Characteristics of participants

The summary characteristics of participants are presented in Table 1. In total, 30 participants were interviewed. Out of the 30 participants, more than half (n = 17) had been vaccinated against COVID-19. The age range was 21 to 67 years. Participants who had been vaccinated against COVID-19 were older (42.3 years) than those who had not been vaccinated (31.8 years). All participants had formal education. Specifically, more than half of participants (n = 16) had attained tertiary education while one-tenth (n = 3) had attained primary education. All participants were Christians, and the majority of them (n = 29) resided in urban areas. All participants who had not been vaccinated against COVID-19 resided in urban areas. Also, one-third of participants (n = 10) were married, while half (n = 15) were never married. Three out of every ten participants were services and sales workers, while a little over two-fifth (n = 12) were professionals. Most participants who had not been vaccinated against COVID-19 were professionals (n = 7), while most participants who had been vaccinated were services and sales workers (n = 7). Furthermore, a higher proportion of participants (n = 14) had no children, while a third (n = 10) had between 1 and 2 children. Fifteen participants were each interviewed in the Greater Accra and Ashanti region. Most participants (n = 22) had no history of non-communicable disease. However, five participants were diagnosed with hypertension, while two were diagnosed with stomach ulcers.

**Table 1:** Socio-demographic characteristics of participants.

Characteristics	Number of Participants Vaccinated (N = 17)	Percentage	Number of Participants Not vaccinated (N = 13)	Percentage	Total Number of Participants (N = 30)	Percentage
**Age**						
Range	21–67		20–56		20–67	
Mean	42.3		31.8		37.7	
Standard Deviation	13.8		9.4		13.0	
**Education**						
Primary	3	17.6	0	0.0	3	10.0
Senior High	6	35.3	5	38.5	11	36.7
Tertiary	8	47.1	8	61.5	16	53.3
**Marital status**						
Never married	7	41.2	8	61.5	15	50.0
Married	5	29.5	5	38.5	10	33.3
Separated	1	5.9	0	0.0	1	3.3
Divorced	2	11.7	0	0.0	2	6.7
Widow	2	11.7	0	0.0	2	6.7
**Religion**						
Christian	17	100.0	13	100.0	30	100.0
**Occupation**						
Administrator	0	0.0	1	7.7	1	3.3
Professional	5	29.4	7	53.8	12	40.1
Services and sales worker	7	41.1	2	15.4	9	30.1
Student	1	5.9	3	23.1	4	13.3
Retired	1	5.9	0	0.0	1	3.3
National service personnel	1	5.9	0	0.0	1	3.3
Seamstress	1	5.9	0	0.0	1	3.3
Housewife	1	5.9	0	0.0	1	3.3
**Number of children**						
0	6	35.3	8	61.5	14	46.7
1–2	8	47.1	2	15.4	10	33.3
3–4	2	11.7	2	15.4	4	13.3
5 and above	1	5.9	1	7.7	2	6.7
**Place of residence**						
Urban	16	94.1	13	100.0	29	96.7
Rural	1	5.9	0	0.0	1	3.3
**Region of residence**						
Greater Accra	10	58.8	5	38.5	15	50.0
Ashanti	7	41.2	8	61.5	15	50.0
**History of non-communicable disease**						
None	12	70.6	10	76.9	22	73.3
Hypertension	4	23.5	1	7.7	5	16.7
Stomach ulcer	0	0.0	2	15.4	2	6.7
Sickle cell disease	1	5.9	0	0.0	1	3.3

### Results from thematic analysis

The thematic findings are presented under two main themes: (a) facilitators of COVID-19 vaccine uptake and (b) barriers to COVID-19 vaccine uptake.

#### Facilitators of COVID-19 vaccine uptake

The facilitators of COVID-19 vaccine uptake were discussed under two main domains: interpersonal and structural factors.

*Interpersonal factors that influence COVID-19 vaccine uptake*. The main interpersonal factors that facilitated the uptake of COVID-19 vaccine among women were the desire to protect oneself and family against COVID-19, and seeing others receive the COVID-19 vaccine.

*Desire to protect oneself and family against COVID-19*. The participants reported that they were motivated to vaccinate against COVID-19 because they wanted to protect their lives and loved ones, including husbands, children, and siblings. They also expressed that they feared getting the virus because they had heard some negative experiences from friends who contracted the diseases and thus, do not wish to go through the same experiences. They further explained that a woman would bear the most brunt when a household member is infected with COVID-19 because of her caregiving responsibilities.

“*My motivation for going for the vaccine is that I have a child*, *siblings and a husband*, *so I do not want them to get COVID-19 because of me*, *so I have to go for the vaccine to protect myself and my family*. *A woman would want to protect her home because when a member of the household is infected with COVID-19*, *she is the one who will suffer most*.*” (R24)*

*“Few of my friends had contracted it [COVID-19], and they told me about their experiences. They said they didn’t wish it on anybody. Also, in the news reports, people narrate what you will experience when you contract the virus. Those accounts made me fear getting COVID-19, which motivated me to get the vaccine.” (R16)*

*Seeing others receive the COVID-19 vaccine*. The participants reported that seeing adult children, in-laws, neighbours, other women, and prominent persons in the country (including the former and current Presidents and their wives) receive their COVID-19 vaccines motivated them to go for their vaccines.

*“The President [of Ghana, Nana Addo Akufo-Addo] got vaccinated. Former President [John Dramani Mahama] got vaccinated, and some of the prominent people did, and a lot of us followed suit. My son also got vaccinated, and my daughter-in-law [a nurse] advised me to go for the vaccine. So, that motivated me to get vaccinated. They set an example for me to follow.” (R12)*

A participant narrated that she was motivated to receive her COVID-19 vaccine after seeing an improvement in the health of her mother and other women after they were vaccinated. She explained that her mother had constant phlegm, but it stopped when she was vaccinated.

“***Participan****t*: *My mum got vaccinated*, *and I saw an improvement in her health*. *For example*, *the cessation of the constant release of phlegm*. *So that motivated me to get vaccinated as well*.

***Interviewer**: So, do you think that her phlegm ceased due to the vaccine she received?*

***Participant**: Yes, because she has not experienced it again since then. A woman at the hospital also testified that her blood wasn’t able to flow well in her left leg, but after being vaccinated, everything went back to normal. So, she also motivated me. So, when women meet, share our positive experiences with others, it can encourage them to get vaccinated.” (R14)*

Another participant reported that women would be encouraged to vaccinate if fellow women who have been fully vaccinated against COVID-19 shared their vaccination experiences. Also, such women could be used as role models to encourage women to be vaccinated against COVID-19.

“*You can bring in old women or women in the community or other places who have taken all the two jabs*, *especially to talk to them that “I took the vaccine and nothing happened to me”*. *This will encourage women to go for the vaccine*, *especially those breastfeeding*. *I think the only people who cannot go for the vaccines are the pregnant women*, *so you can ask the breastfeeding mothers who have gone for their vaccine to talk to other breastfeeding mothers to encourage them to go for theirs*.*” (R13)*

*Structural facilitators of COVID-19 vaccine uptake*. The participants expressed that education about COVID-19 vaccines, and vaccine being cost-free mainly facilitated COVID-19 vaccine uptake. Other structural facilitators included receiving a vaccination card, and giving souvenirs to vaccinated persons.

*Education about COVID-19 vaccines*. The participants highlighted that comprehensive education about the COVID-19 vaccines, specifically the chemicals used in manufacturing them and their side effects, will motivate the vaccine uptake. The participants further stated that education about the COVID-19 vaccine helped to demystified misconceptions about the COVID-19 vaccines and inspired them to go for the vaccines.

“*Education about the COVID-19 vaccines motivated me to go for it*. *I was educated on the side effects and what the vaccine would do in my system*. *The vaccine was going to reduce the seriousness or the severity of the disease [COVID-19] should I be affected*.” (R11)

One participant elaborated that she was satisfied after reading about the COVID-19 vaccine and its benefits, so she decided to vaccinate.

*“I read about the dos and don’ts of the [COVID-19] vaccine and whether it will be helpful for persons who go for them*. *I was satisfied with what I read*, *so I decided to go for the vaccination*.*”* (R13)

*Vaccine being cost-free*. The provision of COVID-19 vaccines free of charge to citizens was identified as a facilitator of COVID-19 uptake among the women. Women were encouraged to go for COVID-19 vaccines because they didn’t pay for them.

*“Ghanaians like free things. If we have to pay for the vaccines, it will just be a few people who will go for it, but once it’s free, you see the masses go for it.” (R4)*

*“The COVID-19 vaccine is cost-free, so I vaccinated. It’s also a motivating factor why women will take it.” (R11)*

*Receiving a vaccination card*. The vaccination cards issued to clients after vaccination emerged as a motivation for the uptake of COVID-19 vaccines. Some participants anticipate that the vaccination card will be required to have access to social services (such as banks), seek employment, and to travel abroad. The need to avoid these restrictions will motivate women to get vaccinated.

*“If you don’t inject the vaccine, you cannot get the vaccination card. If you don’t get that vaccination card, there are certain places you cannot go, like travelling outside the country. Because people want the vaccination card, they go for the vaccination. (R4)*

*“COVID-19 has come to stay with us like AIDS, so I think a time will come that people wouldn’t be able to go to the bank, even travel outside the country, or the card will be a prerequisite for employment. So that motivated me to get the COVID-19 vaccine.” (R29)*

*Giving souvenirs to vaccinated persons*. Souvenirs refer to gifts given to someone in cash or in-kind. Participants expressed that souvenirs in cash or in-kind (such as food) can encourage women to get vaccinated, especially those in rural and urban resource-poor areas.

*“You know people like gifts, so if you give them things, especially those in the rural areas or those in urban resource-poor areas, it will attract them to buy into the idea of vaccinating themselves against COVID-19.” (R16)*

#### Barriers to COVID-19 vaccine uptake

Three-organising themes were discernible under barriers to COVID-19 vaccine uptake–individual, interpersonal and structural barriers

*Individual level barriers to COVID-19 vaccine uptake*. The dominant individual level barrier to the uptake of COVID-19 vaccines was fear of side effects. Tight work schedules, vaccine effectiveness, and pregnancy emerged as other individual barriers to COVID-19 vaccine uptake.

*Fear of side effects*. The side effects (example: feeling chills, difficulty lifting arm that was injected, headaches, and tiredness) experienced by people who took the COVID-19 vaccine prevented some participants from vaccinating against COVID-19. That is, they fear experiencing similar sides effects.

*“Some women are afraid to go for the COVID-19 vaccination because those who went for the vaccination came back complaining about a headache, feeling feverish, and difficulty raising their arms.” (R3)*

*Initially, I wanted to go for the COVID-19 vaccination because I perceived it was needful. But as time went on, people vaccinated against COVID-19 started to experience side effects. The side effects they experienced killed my enthusiasm.” (R25)*

*Tight work schedule*. Some participants explained that their tight work schedule hindered their participation in the COVID-19 vaccination exercise. They revealed that they go to work early and close late, and hence they are unable to go to the vaccination centres for the vaccine.

*“I go to work by 7:00 am and close by 5:00 pm. By the time I close from work, health workers at the vaccination centres would have also close. Even if I would like to go for the COVID-19 vaccine before going to work, health workers at the vaccination centres start work at 8:00 am, and I can only go there before 7:00 am.” (R22)*

*Effectiveness of the vaccines*. The participants explained that the perceived effectiveness of the COVID-19 vaccine would affect the participation of women in the exercise. This implies that the COVID-19 vaccine being effective would encourage women to vaccinate. However, because the COVID-19 vaccines are cost-free, some people perceive them as ineffective. They also explained that some people associate the effectiveness of a vaccine with the country of origin. For example, Johnson and Johnson COVID-19 vaccines are perceived as effective than AstraZeneca vaccines because the former was manufactured in the United States of America (USA). In contrast, the latter was manufactured in India.

*“People [women] think since the COVID-19 vaccine is cost-free, it is not good. …. Also, the Johnson and Johnson vaccine is from the United States of America (USA), so it is good. The AstraZeneca vaccine is from India, so it is not good.” (R13)*

*Pregnancy*. Some participants narrated that a woman’s pregnancy status may influence her participation in the COVID-19 vaccination exercise. They explained that pregnant women are exempted from taking the vaccine. They also revealed that pregnancy is sacred, so pregnant women will not vaccinate since they are uncertain about the impact of the vaccine on their fetuses.

*“You know pregnancy is very sacred, so if pregnant women are not sure how it will affect the fetus, they will not go for the COVID-19 vaccine.” (R16)*

*Interpersonal level barriers to COVID-19 vaccine uptake*. Misconceptions about COVID-19 merged as the main interpersonal barrier to the uptake of COVID-19 vaccines.

*Misconceptions about the vaccines*. The participants reported that some women perceive that being injected with COVID-19 vaccines will cause barrenness in women, impotency in men, make people foolish/stupid, and the vaccine is meant to kill Africans, including Ghanaians.

*“Some people have the perception that the Whites [Europeans and Americans] want to eliminate Ghanaians from the world, so they have intentionally brought the vaccine so that in some years to come, all of us will die, and they will take over the country. Some are also of the view that it will make the men impotent, and when women vaccinate, they will not be able to conceive a child because it will destroy their ovaries. Others are also of the view that it’s going to make us foolish/stupid so that we will follow whatever the whites tell us to do.” (R11)*

Some participants highlighted that some pastors admonished their congregants not to accept the COVID-19 vaccine since it is demonic/satanic. Instead of the vaccines, these pastors gave their congregants spiritual directions to help protect them against COVID-19.

*“Some people [women] have also been told by their pastors that the COVID-19 vaccine is demonic, so they shouldn’t go for the vaccine. I have witnessed one. The pastor of my aunt’s church has advised the congregation against taking the COVID-19 vaccine. So, I think some of the pastors also discourage people from going for the vaccines.” (R13)*

*Structural barriers to COVID-19 vaccine uptake*. Participants identified long queues at hospitals, health centres or vaccination centres, and shortage of vaccines as the main structural barriers to COVID-19 vaccine uptake. In addition, proximity to a hospital, health centre, or vaccination centre was mentioned as another structural barrier to COVID-19 vaccine uptake.

*Long queues at hospitals*, *health centres or vaccination centres*. The participants elaborated that long queues at hospitals, health centres or vaccination centres prevented women, including themselves, from getting their COVID-19 vaccine. They also acknowledged that when one joins a long queue, there is no guarantee the person will be vaccinated that same day. Consequently, people sometimes sleep at vaccination centres to avoid long queues.

*“There were long queues in some communities, and it was a hindrance for people to get vaccinated because nobody would want to go [a hospital, health centre or vaccination centre] and waste their time there.” (R27)*

*“Some people even sleep at the vaccination centres in the name of forming a queue*.*”* (R28)

*Shortage of vaccines*. Shortage pertains to an inadequate supply of vaccines in relation to demand. Inadequate supply at hospitals, health centres or vaccination centres prevented women from receiving the COVID-19 vaccines. This implies that making COVID-19 vaccines available and accessible to women would encourage COVID-19 vaccination. Participants explained that people devised a strategy of sleeping at hospitals, health centres or vaccination centres to ensure they get vaccinated.

*“When I went for my vaccination against COVID-19, they [health workers] said the first supply has finished and the second supply was for those who had received the first dose. That is why I haven’t been vaccinated.” (R10)*

*“Currently, there is a shortage of the [COVID-19] vaccines. People are willing to get vaccinated, but there are no vaccines available.” (R24)*

*Proximity to hospital*, *health centre or vaccination centre*. The closeness of an individual to a health facility or vaccination centre may influence his/her utilisation of the health facility or vaccination centre. Participants expressed that their inability to go for the vaccine could be attributed to the distance between their place of residence and health facilities or vaccination centres. Therefore, the proximity of women to hospitals, health and vaccination centres served as a motivation for them to get vaccinated.

*“One factor that will discourage me from going for the coronavirus injection is the distance I will have to take before going for the injection. If it is close by, then I will go for it, but if it is not, then I will not go for it.” (R3)*

## Discussion

In the fight against the COVID-19 pandemic, population-wide vaccination is still regarded as one of the most effective tools in the public health toolkit. The present study critically examined the facilitators and barriers to COVID-19 vaccine uptake among women in two regions of Ghana using semi-structured qualitative interviews. The discussion is organised under (a) facilitators of COVID-19 vaccines uptake and (b) barriers to COVID-19 vaccines uptake.

### Facilitators of COVID-19 vaccines uptake

The desire to protect oneself and family from contracting COVID-19 and seeing others receive the COVID-19 vaccine were the key interpersonal level facilitators of COVID-19 vaccine uptake among women. Because COVID-19 is an infectious disease with a high infection fatality rate [32], most participants took precautions to avoid contracting it and transmitting it to others. This finding is consistent with the results of Agyekum et al. [16], who found that a higher proportion of respondents who had come into contact with a COVID-19 patient, as well as those who had a family member or friend diagnosed with COVID-19, indicated acceptance of COVID-19 vaccines. Similarly, Acheampong et al. [20] found that the desire to protect oneself, family, friends, and community against COVID-19 were the main reasons for respondents who are ‘very likely’ or ‘somewhat likely’ to take the COVID-19 vaccines.

Also, the finding showed that seeing others including adult children, neighbours, other women, and prominent people in the country receive their COVID-19 vaccines motivated women to get vaccinated. This highlights the crucial role significant others play in shaping individuals’ actions and behaviours. Prior to Ghana receiving its first dose of the AstraZeneca/Oxford vaccine via the COVAX facility, there were many misconceptions about the vaccine’s efficacy and side effects, which had the potential to increase hesitancy and reduce uptake. To allay Ghanaians’ fears, build social trust, and accept the vaccines, the President of Ghana, his Vice, their respective spouses, and some ministers of state took the initiative to be the first to get vaccinated [33]. Also, female celebrities were appointed as COVID-19 vaccination ambassadors to encourage COVID-19 vaccine uptake [34]. The vaccination of these prominent figures, which was broadcast live on television, significantly reduced public fear, giving Ghana’s vaccination programme a good start. Studies have found that role models and peers are effective strategies to increase vaccine uptake [35–38].

At the structural level, the participants reported that education about the side effects of the COVID-19 vaccines and ingredients used in manufacturing the vaccines facilitated its uptake among women. This view suggests shortfalls in the ongoing educational campaigns to whip up interest in getting vaccinated, hence, the call for comprehensive education to enhance their knowledge on COVID-19. Research has established that education on vaccination, specifying the vaccine safety, possible side effects, and benefits influence vaccine uptake [39]. In Ghana, the Ghana Health Service (GHS) regularly held press briefings to provide a situational report on COVID-19 and educate the populace about the COVID-19 vaccine. Also, health personnel involved in the vaccination programme sensitised the general public about the safety, efficacy, possible side effects and benefits of receiving the COVID-19 vaccine, which has aided in the acceptance and uptake of the vaccines. However, as highlighted by participants, such press briefings and education have reduced over time. This finding implies a need to repackage COVID-19 information and dissemination to address the needs of the different segments of the population.

In addition, COVID-19 vaccines being cost-free motivated participants and other women to participate in the vaccination programme. This finding is similar to studies in France [40], Spain [41], the United States of America [42], which found that free vaccination increases the uptake of vaccination. Similarly, a systematic review by Yeung et al. [43] found that free vaccination increases vaccination uptake. Affordability is a significant concern in health care delivery and, by extension, vaccination/immunization programmes [44–46]. Given that one of the primary reasons for vaccine hesitancy is a lack of affordability, it is not surprising that the women who participated in this study indicated that providing the COVID-19 vaccines for free would encourage other women to get vaccinated.

### Barriers to COVID-19 vaccines uptake

Vaccine hesitancy can pose a significant threat to global public health by contributing to suboptimal vaccine coverage. Vaccine hesitancy is likely due to several factors; however, the 5C model relates vaccine hesitancy to confidence, convenience (or constraints), complacency, calculated risk, and collective responsibility [12]. In this study, fear of side effects such as chills, difficulty lifting the injected arm, headaches, and tiredness were reported as the main individual level barrier to COVID-19 vaccine uptake. This relates with calculated risk in the 5C model. Studies have shown that side effects such as chills, fever, and swelling of lymph are typical of vaccines because an introduction of a foreign substance into the body elicits an immune response [47,48]. However, not all individuals may exhibit these side effects [49]. In addition, because these sides effects are unpleasant, some individuals may misconstrue them as sickness and perceive the COVID-19 vaccine as disease-causing [49,50]. Therefore, education by public health professionals on the side effects of vaccine uptake and remedies to alleviate these side effects in women can increase their uptake.

Congruent with studies elsewhere [51–53], misconceptions about COVID-19 were reported as one of the main barriers to COVID-19 vaccines acceptance in the present finding. From the narratives, there were misconceptions that COVID-19 vaccines were demonic/satanic, could cause infertility in both men and women and it is meant to kill Africans, including Ghanaians. Tamysetty et al.’s [54] study in India reported similar misconceptions, including the belief that the COVID-19 vaccine could increase the risk of death and decrease a woman’s ability to conceive children. Studies have also documented that some Christians associate the COVID-19 vaccine with the mark of the beast/666 [55,56]. Instead of advocating for vaccine uptake, these religious leaders provided spiritual guidance to their congregations to help protect them from COVID-19. Such guidelines and misconceptions about COVID-vaccine negatively affect its uptake [57–59]. We recommend that further studies should employ quantitative approaches to examine the prevalence of the misconceptions identified in this study and how these are likely to influence vaccination generally. Such information might guide public health professionals in rolling out effective methods for vaccinations among Ghanaians with misconceptions about vaccines.

The findings also identified long queues at hospitals, health centres, or vaccination centres as the primary structural barrier that prevented women, including the participants, from receiving their COVID-19 vaccination. Worryingly, joining a long queue did not guarantee one to receive vaccination for that day. Although, Ghana used outreach points, mobile clinics, markets and other public places to supplement its limited number of hospitals and vaccination centres to carry out the vaccination programme [60], the number of people visiting these facilities to get vaccinated was enormous. It, consequently, resulted in long wait times in queues. Consistent with our finding, the uncertainty of not getting vaccinated and the nature of the registration process have been reported among women and older people as barriers against COVID-19 vaccination [54].

Another structural barrier reported by the participants was shortage of vaccines. This implies that making COVID-19 vaccines available and accessible to women would encourage COVID-19 vaccination. In Ghana, there were delays in receiving the second dose of the AstraZeneca/Oxford vaccine [61] during the initial enrollment of the vaccination programme when demand for vaccine uptake was high. This therefore affected uptake.

A tight work schedule was also identified as a barrier to the uptake of COVID-19 vaccination. Participants whose work schedules required them to leave early and return late were unable to participate in the COVID-19 vaccination programme. Institutions or organizations should negotiate with health facilities to bring vaccines to their workplaces to increase the uptake of the vaccines among their workers. Given that several COVID-19 vaccines are being rolled out at an unprecedented rate and scale, gender considerations must be prioritized throughout the vaccine deployment process.

### Limitation

There are some limitations to this study. In particular, the qualitative method employed does not permit generalisation of the results to the entire women population in Ghana. Also, all the study participants were Christians and had formal education. However, these characteristics of our participants are not representative of women’s attributes in Ghana. In Ghana, the majority of women are Christians (79%) and formally educated (83%) [62]. Therefore, our findings should be interpreted with caution. Despite these limitations, this study is timely and critical for two key reasons. First, using a qualitative method allowed participants’ reasoning to be deeply explored, thereby providing a richness of insights that quantitative survey strategies have been missing. Secondly, for a crucial and nuanced issue, such as COVID-19 vaccine uptake, the ability to probe and explore participants’ opinions on facilitators and barriers is vital, particularly as governments and public health practitioners continue to strategize how best to push further COVID-19 vaccine acceptance and uptake.

## Conclusions

This study demonstrates that individual, interpersonal and structural factors facilitate or hinder COVID-19 vaccination among women. The findings provide nuanced information to inform relevant stakeholders involved in the COVID-19 vaccination programme in Ghana and beyond, to put in place measures to amplify efforts towards sustaining or improving the facilitators identified to increase uptake and address the barriers to reduce hesitancy among women. Addressing these factors need continuous comprehensive health education, and ensuring vaccine availability at hospitals, health, and vaccination centres will improve women’s uptake of the COVID-19 vaccines.

## Supporting information

S1 Checklist COREQ (COnsolidated criteria for REporting Qualitative research) checklist. (PDF)

S1 File Interview guide administered to women. (DOCX)

S1 Appendix Themes for facilitators and barriers to COVID-19 vaccine uptake among women. (DOCX)
