# Supplementary material for: Facilitators and barriers to COVID-19 vaccine uptake among women in two regions of Ghana: A qualitative study
Source: PLoS One. 2022 Aug 17;17(8):e0272876. doi: 10.1371/journal.pone.0272876 (PMC9385066; doi:10.1371/journal.pone.0272876)
Supplement: S1 File — (DOCX) [file pone.0272876.s002.docx]

**Interview guide administered to women**

**Background characteristics**

1. Can you tell me something about yourself?

- Probe: Age, Educational background, Occupation, Marital status, Religion, Number of children, Place of residence (Urban/Rural) etc.

1. Have you been diagnosed with any health condition(s)?

- Probe for chronic non-communicable conditions. E.g., hypertension, diabetes, asthma, heart conditions, etc.

**COVID-19 experience**

1. Have you ever tested for COVID-19?

- If yes, what was the results?
- If no, would you take a test if you were offered one for free? Why?

1. If you have ever tested positive, what treatment did you receive or do?

- Probe for pluralistic health seeking

**COVID-19 vaccination**

1. What do you know about COVID-19 vaccination?
2. What are the general perceptions about COVID-19 vaccines?
3. What is your perception about COVID-19 vaccines?
4. Have you been vaccinated for COVID-19?
5. If vaccinated, when did you get vaccinated?
6. How many dose(s) have you taken/received?
7. Can you tell me your experience after receiving your vaccine?

- Probe: Side effect
- Probe: Did you feel safe and why?
- Probe: Regret after taking the vaccine

1. How did you manage your experience? (Ask if respondent had a negative experience. E.g. experienced side effect(s)

**Motivation to accept COVID-19 vaccine**

1. What factor(s) do you think would motivate women to go for the COVID-19 vaccine?

- Probe: Fear of contracting the disease
- Probe: Safety of the vaccine
- Probe: Effectiveness/efficacy of the vaccine
- Probe: Government’s directives
- Probe: Accessibility of the vaccine
- Probe: Vaccine being free
- Probe: Having underlying health condition(s)
- Probe: Media influence

1. What factor(s) would motivate you to go for the COVID-19 vaccine?

- Probe: Fear of contracting the disease
- Probe: Safety of the vaccine
- Probe: Effectiveness/efficacy of the vaccine
- Probe: Government’s directives
- Probe: Accessibility of the vaccine
- Probe: Vaccine being free
- Probe: Having underlying health condition(s)
- Probe: Media influence

**Barriers to accept COVID-19 vaccine**

1. What factor(s) would prevent women to go for the COVID-19 vaccine?

- Probe: The disease does not exist
- Probe: Shortage of vaccine
- Probe: Safety of the vaccine
- Probe: Effectiveness/efficacy of the vaccine
- Probe: No health condition
- Probe: Media influence
- Probe: Distance to health facility for the vaccine
- Probe: Queue for the vaccine
- Probe: Cultural belief

1. What factor(s) would prevent you to go for the COVID-19 vaccine?
2. What advice would you give to women who are yet to vaccinate against COVID-19?
