# Supplementary material for: Facilitators and barriers to COVID-19 vaccine uptake among women in two regions of Ghana: A qualitative study
Source: PLoS One. 2022 Aug 17;17(8):e0272876. doi: 10.1371/journal.pone.0272876 (PMC9385066; doi:10.1371/journal.pone.0272876)
Supplement: S1 Appendix — (DOCX) [file pone.0272876.s003.docx]

**Themes for facilitators and barriers to COVID-19 vaccine uptake among women**

| **Global themes** | **Organising themes** | **Basic themes** | **Sample quotes** |
| --- | --- | --- | --- |
| Facilitators of COVID-19 vaccine uptake | Interpersonal facilitators of COVID-19 vaccine uptake | Desire to protect oneself and family against COVID-19 | “I do not want to contract the virus or perhaps even if I should contract it, I don’t want to go through any severe issues.” (R15)  “Few of my friends had contracted it [COVID-19], and they told me about their experiences. They said they didn’t wish it on anybody. Also, in the news reports, people narrate what you will experience when you contract the virus. Those accounts made me fear getting COVID-19, which motivated me to get the vaccine.” (R16)  “My motivation for going for the vaccine is that I have a child, siblings and a husband, so I do not want them to get COVID-19 because of me, so I have to go for the vaccine to protect myself and my family. A woman would want to protect her home because when a member of the household is infected with COVID-19, she is the one who will suffer most.” (R24) |
|  |  | Seeing others receive the COVID-19 vaccine | “The President [of Ghana, Nana Addo Akufo-Addo] got vaccinated. Former President [John Dramani Mahama] got vaccinated, and some of the prominent people did, and a lot of us followed suit. My son also got vaccinated, and my daughter-in-law [a nurse] advised me. So that motivated me to get vaccinated. They set an example for me to follow.” (R12)  “Participant: My mum got vaccinated, and I saw an improvement in her health. For example, the cessation of the constant release of phlegm. So that motivated me to get vaccinated as well.  Interviewer: So, do you think that her phlegm ceased due to the vaccine she received?  Participant: Yes, because she has not experienced it again since then. A woman at the hospital also testified that her blood wasn’t able to flow well in her left leg, but after being vaccinated, everything went back to normal. So, she also motivated me. So, when women meet, share our positive experiences with others, which can encourage them to get vaccinated.” (R14)  “You can bring in old women or women in the community or other places who have taken all the two jabs, especially to talk to them that “I took the vaccine and nothing happened to me”. This will encourage women to go for the vaccine, especially those breastfeeding. I think the only people who cannot go for the vaccines are the pregnant women, so you can ask the breastfeeding mothers who have gone for their vaccine to talk to other breastfeeding mothers to encourage them to go for theirs.” (R13) |
|  | Structural facilitators of COVID-19 vaccine uptake | Education about COVID-19 vaccines | “Education about the COVID-19 vaccines motivated me to go for it. I was educated on the side effects and what the vaccine would do in my system. The vaccine was going to reduce the seriousness or the severity of the disease [COVID-19] should I be affected.” (R11)  “I read about the dos and don’ts of the [COVID-19] vaccine and whether it will be helpful for persons who go for them. I was satisfied with what I read, so I decided to go for the vaccination.” (R13) |
|  |  | Vaccine being cost-free | “Ghanaians like free things. If we have to pay for the vaccines, it will just be a few people who will go for it, but once it’s free, you see the masses go for it.” (R4)  “The COVID-19 vaccine is cost-free, so I vaccinated. It’s also a motivating factor why women will take it.” (R11) |
|  |  | Getting vaccination card | “If you don’t inject the vaccine, you cannot get the vaccination card. If you don’t get that vaccination card, there are certain places you cannot go, like travelling outside the country. Because people want the vaccination card, they go for the vaccination. (R4)  “COVID-19 has come to stay with us like AIDS, so I think a time will come that people wouldn’t be able to go to the bank, even travel outside the country, or the card will be a prerequisite for employment. So that motivated me to get the COVID-19 vaccine.” (R29) |
|  |  | Giving souvenirs to persons who vaccinate | “You know people like gifts, so if you give them things, especially those in the rural areas or those in urban resource-poor areas, it will attract them to buy into the idea of vaccinating themselves against COVID-19.” (R16) |
| Barriers to COVID-19 vaccine uptake | Individual barriers to COVID-19 vaccine uptake | Fear of side effects | “Some women are afraid to go for the COVID-19 vaccination because those who went for the vaccination came back complaining about a headache, feeling feverish, and difficulty raising their arms.” (R3)  Initially, I wanted to go for the COVID-19 vaccination because I perceived it was needful. But as time went on, people vaccinated against COVID-19 started to experience side effects. The side effects they experienced killed my enthusiasm.” (R25) |
|  |  | Tight work schedule | “I go to work by 7:00 am and close by 5:00 pm. By the time I close from work, health workers at the vaccination centres would have also close. Even if I would like to go for the COVID-19 vaccine before going to work, health workers at the vaccination centres start work at 8:00 am, and I can only go there before 7:00 am.” (R22) |
|  |  | Vaccine effectiveness | “People [women] think since the COVID-19 vaccine is cost-free, it is not good. …. Also, the Johnson and Johnson vaccine is from the United States of America (USA), so it is good. The AstraZeneca vaccine is from India, so it is not good.” (R13) |
|  |  | Pregnancy | “You know pregnancy is very sacred, so if pregnant women are not sure how it will affect the fetus, they will not go for the COVID-19 vaccine.” (R16) |
|  | Interpersonal barriers to COVID-19 vaccine uptake | Misconceptions about the vaccines | “Some people have the perception that the Whites [Europeans and Americans] want to eliminate Ghanaians from the world, so they have intentionally brought the vaccine so that in some years to come, all of us will die, and they will take over the country. Some are also of the view that it will make the men impotent, and when women vaccinate, they will not be able to conceive a child because it will destroy their ovaries. Others are also of the view that it’s going to make us foolish/stupid so that we will follow whatever the whites tell us to do.” (R11)  “Some people [women] have also been told by their pastors that the COVID-19 vaccine is demonic, so they shouldn’t go for the vaccine. I have witnessed one. The pastor of my aunt’s church has advised the congregation against taking the COVID-19 vaccine. So, I think some of the pastors also discourage people from going for the vaccines.” (R13)  “The last time, I went to the saloon and one lady objected that she won’t get vaccinated against COVID-19 because her pastor has given them some spiritual directions to follow. Others also revealed that their pastors have done spiritual stuff for them so COVID-19 cannot infect them so they should not go for any COVID-19 vaccines.” (R14)  “I heard people saying that when a woman takes the COVID-19 vaccine, she wouldn’t be able to give birth because it will destroy her ovaries and that prevents women from going for the vaccines.” (R28) |
|  | Structural barriers to COVID-19 vaccine uptake | Long queues at hospitals, health centres or vaccination centres | “The long queues and how the [vaccination] centre was overcrowded with people made me to keep procrastinating it.” (R21)  “There were long queues in some communities, and it was a hindrance for people to get vaccinated because nobody would want to go [a hospital, health centre or vaccination centre] and waste their time there.” (R27)  “Some people even sleep at the vaccination centres in the name of forming a queue.” (R28) |
|  |  | Shortage of vaccines | “When I went for my vaccination against COVID-19, they [health workers] said the first supply has finished and the second supply was for those who had received the first dose. That is why I haven’t been vaccinated.” (R10)  “Currently, there is a shortage of the [COVID-19] vaccines. People are willing to get vaccinated, but there are no vaccines available.” (R24) |
|  |  | Proximity to a hospital, health centre or vaccination centre | “One factor that will discourage me from going for the coronavirus injection is the distance I will have to take before going for the injection. If it is close by, then I will go for it, but if it is not, then I will not go for it.” (R3)  “If the COVID-19 vaccines are available and it is not close to my community, I wouldn’t go for it.” (R6) |
